# Tumor-Infiltrated CD8+ T Cell 10-Gene Signature Related to Clear Cell Renal Cell Carcinoma Prognosis

**DOI:** 10.3389/fimmu.2022.930921

**Published:** 2022-06-24

**Authors:** Jie Wang, Feifan Huang, Jingjie Zhao, Peng Huang, Junhua Tan, Meiying Huang, Ruiying Ma, Yu Xiao, Siyuan He, Zechen Wang, Jiajia Shen, Heming Lu, Lingzhang Meng

**Affiliations:** ^1^Center for Systemic Inflammation Research (CSIR), Youjiang Medical University for Nationalities, Baise, China; ^2^Department of Kidney Diseases, Affiliated Hospital of Youjiang Medical University for Nationalities, Baise, China; ^3^Life Science and Clinical Research Center, Affiliated Hospital of Youjiang Medical University for Nationalities, Baise, China; ^4^Department of Radiation Oncology, People’s Hospital of Guangxi Zhuang Autonomous Region, Nanning, China; ^5^Institute of Cardiovascular Sciences, Guangxi Academy of Medical Sciences, Nanning, China

**Keywords:** scRNA-seq, clear cell renal cell carcinoma, papillary renal cell carcinoma, CD8+ T cell, neutrophil metabolism

## Abstract

Clear cell renal cell carcinoma (ccRCC) usually affects multiple organs (e.g., bone and brain), and patient prognosis is usually poor. Although it is known that CD8+ T cell infiltration can potentially alleviate ccRCC progression, few studies have concentrated on the correlation between CD8+ T cell infiltration and ccRCC prognosis. In this study, ten genes expressed by infiltrated CD8+ T cells (i.e., *AMD1, CCSER2, CIB1, DRAP1, HMGB2, HMGN1, NPIPB5, PTP4A2, RORA*, and *SAP18*) were suggested as potential ccRCC prognostic biomarkers, by using next-generation sequencing (i.e. bulk sequencing and single-cell sequencing) of ccRCC, papillary renal cell carcinoma (papRCC), and control kidney biopsies. Specifically, we identified four genes (i.e., *CCSER2, DRAP1, NPIPB5*, and *SAP18*) as potential novel prognostic biomarkers for ccRCC. It is noteworthy that *SAP18* derived from CD8+ T cells negatively correlates to Atg7+ neutrophils in ccRCC, compared with papRCC, indicating a potential decreased neutrophil metabolic function in autophagy and fatty acids. This study elucidated the protective role of infiltrated CD8+ T cells in ccRCC and identified ten candidate genes related to an improved prognosis in patients with ccRCC.

## Introduction

Clear cell renal cell carcinoma (ccRCC) is a malignant cancer affecting the urine system (1). It accounts for more than 80% of kidney cancers (2), and is known for its high mortality rate. The lack of adequate prognostic biomarkers for ccRCC lowers clinical treatment efficiency. Although predictive markers for kidney cancers have been extensively explored (3), few studies have focused on ccRCC.

The immune microenvironment determines the clinical therapy and survival of patients with cancer (4). Thus, prognostic biomarkers should be identified within the tumor immune microenvironment. B and natural killer (NK) cells have been reported to correlate with breast cancer prognosis (5, 6) and monocytes and lymphocytes can be used to evaluate the survival of patients with ovarian cancer (7). CD4+ T cell infiltration suggests an improved prognosis for patients with urinary bladder cancer (8). Although infiltrated CD8+ T cells were found to serve as prognostic biomarkers for various cancers (e.g., squamous cell carcinoma, non-small lung carcinoma, and esophageal carcinoma) (9–11), no studies have assessed the potential function of infiltrated CD8+ T cells in the prognosis of patients with ccRCC, to the best of our knowledge.

In this study, infiltrated CD8+ T cells and relevant genes were evaluated as potential predictive biomarkers for ccRCC and papRCC based on bulk sequencing and single cell RNA sequencing (scRNA-seq) data of ccRCC and papRCC biopsies.

## Materials and Methods

### Estimation of Infiltrated CD8+ T Cell in ccRCC Biopsies

The database TIMER2.0 (http://timer.comp-genomics.org/) was used to assess the relationship of infiltrated CD8+ T cells in ccRCC biopsies. This database was designed to compare immune cells among multiple tumor types by performing Cox regression and Kaplan-Meier survival analyses.

### Identification of CD8+ T Cell-Related Genes in ccRCC and papRCC Biopsies

The transcriptome data of ccRCC (n = 3), papRCC (n = 1), and control kidney biopsies were obtained from Young et al. (12). The data were normalized using R package SCTransform, clustered under a resolution of 0.1 and presented with UMAP plots using R package Seurat (https://satijalab.org/seurat/v3.1/integration.html). For quality control, unique molecular identifier (UMI) counts < 500 and doubles were removed. CD8+ T cells were isolated for subsequent analysis.

### scRNA-Seq Analysis of Large-Scale ccRCC Datasets

The datasets for scRNA-seq of 602 ccRCC samples were retrieved from The Cancer Genome Atlas (TCGA) database. The TCGA samples were randomly separated into training and testing groups. The raw gene expression dataset was processed and genes were annotated with a probe ID using R package Bioconductor and compared after normalization and Log2 transformation. Samples with missing clinical information were excluded.

### Selection of CD8+ T Cell-Related Gene Signature

The scRNA-seq data were stratified into cell types based on their genetic profiles, and differentially expressed genes (DEGs) were identified. Thresholds of min.pct > 0.25 and |Log2(FC)| were set. The correlation between CD8+ T cell-related DEGs and ccRCC as well as survival information was presented using the TCGA database. A univariate Cox regression analysis was performed to identify genes associated with survival (p < 0.05). The significance of candidate genes was determined using variable importance (VIMP) within the random survival forest (RSF) algorithm. A multivariate Cox regression analysis was performed to build the risk score model. Receiver operating characteristic (ROC) analysis was conducted to obtain 3-, 5- and 10-year survival rates, and the gene signature’s specificity and sensitivity were assessed by area under the curve (AUC) analysis. In addition, the ccRCC tumor-infiltrated CD8+ T cell-related gene signature was validated to assess the robustness of the outcome.

### Group Investigation

Group analysis was conducted by applying with ccRCC clinical variables to determine the risk score distribution of the relevant genes. Other factors (i.e., age, sex, disease stage, and pathological type) were also analyzed. The prognostic value was calculated by performing a multivariate Cox regression analysis.

### Immunofluorescent Imaging

Cancerous biopsies were isolated from papRCC patients and ccRCC patients under surgery. After embedding with FSC 22 clear solution (Leica, #3801481) and solidified in liquid nitrogen, biopsies were sectioned into 6-μm slides. For fixation, slides were dipped in -20°C methanol for 10 minutes. After washing twice with PBS, samples were blocked with PBS/1%BSA/1%Fcγ blocker at 4°C for 1 hour. Slides were incubated with primary antibodies at 4°C overnight, then with secondary antibodies at room temperature for 1 hour. After mounting with antifade mountant (Beyotime, #P0126). Images were acquired on a fluorescence microscope (Leica DMI3000B).

Antibodies/materials used in this study included: APC rat-anti-human/mouse CD8a (Invitrogen, # 17-0081-82), rabbit-anti-human SAP18 (Invitrogen, # PA5-52821), FITC mouse-anti-human CD66b (Invitrogen, 11-0666-42), rabbit-anti-human ATG7 (Invitrogen, # MA5-32221), Cy3 goat-anti-rabbit IgG (Affinity, #S0011) and DAPI (Invitrogen, D21490).

### Flow Cytometry Analysis

Cancerous biopsies from RCC patients were cut into pieces, and digested with Collagenase IV (15 mg/mL, Gibco, #17104-019) to obtain single cell suspensions. Then the antibody cocktail which contained APC rat-anti-mouse/human CD45 (Invitrogen, # 17-0451-82) and Pacific Orange mosue-anti-human CD8 (Invitrogen, # MHCD0830TR), were used to incubate the cells at 4°C for 15 min, Fixable Viability Dye eFluo 450 (Invitrogen, # 65-0863-14) was used to remove the debris. After washing twice, the counterstained samples were recorded on ThermalFisher Attune NxT machine.

### Statistical Analysis

ROC and Kaplan-Meier survival analyses were performed in combination with “survivor” and “survROC” software packages. The cut-off values were optimized using the “Survminer” package in R software. Univariate and multivariate Cox regression analyses were performed to determine prognostic factors of interest. The software R (v4.0.5) was employed for the above statistics, in which statistical significance was defined as a p-value of < 0.05.

## Results

### CD8+ T Cells Exert Prognostic Value in ccRCC

**Figure 1:**
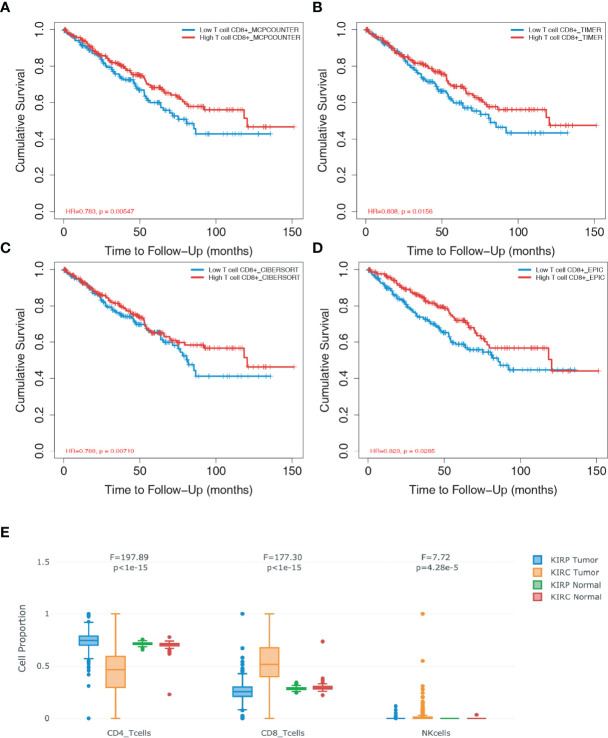
CD8+ T cells exert prognostic value in clear cell renal cell carcinoma (ccRCC). The database TIMER2.0 (http://timer.comp-genomics.org/) was used to assess the relationship of infiltrated CD8+ T cells in ccRCC biopsies. **(A)** Survival analysis from the MCPCOUNTE database indicating the correlation between high infiltration of CD8+ T cells and improved prognosis in patients with ccRCC. **(B)** Survival analysis from the TIMER database showing the correlation between high infiltration of CD8+ T cells and improved prognosis in patients with ccRCC. **(C)** Survival analysis from the CIBERSORT database indicating the correlation between high infiltration of CD8+ T cells and improved prognosis in patients with ccRCC. **(D)** Survival analysis from the EPIC database showing the correlation between high infiltration of CD8+ T cells and improved prognosis in patients with ccRCC. **(E)** Box plots showing the proportion of CD4+ T, CD8+T, and natural killer (NK) cells in ccRCC and papillary renal cell carcinoma (papRCC) biopsies, respectively.

To assess whether CD8+ T cell infiltration affect the prognosis of patients with ccRCC, four databases (i.e., “MCPCOUNTER”, “TIMER”, “CIBERSORT” and “EPIC”) were used for the survival analysis based on the univariate Cox proportional risk model. Follow-up studies on the 10-year cohorts showed that patients with a high level of CD8+ T cells achieved significantly higher survival rates (**Figures 1A–D**), which demonstrated that CD8+ T cells plays a protective role against ccRCC. Compared to patients with papRCC, patients with ccRCC exhibited a significantly higher level of CD8+ T cells (**Figure 1E**), which suggests that the protective role CD8+ T cells could be specific to patients with ccRCC.

### Identification of CD8+ T Cell-Related Genes in ccRCC

**Figure 2:**
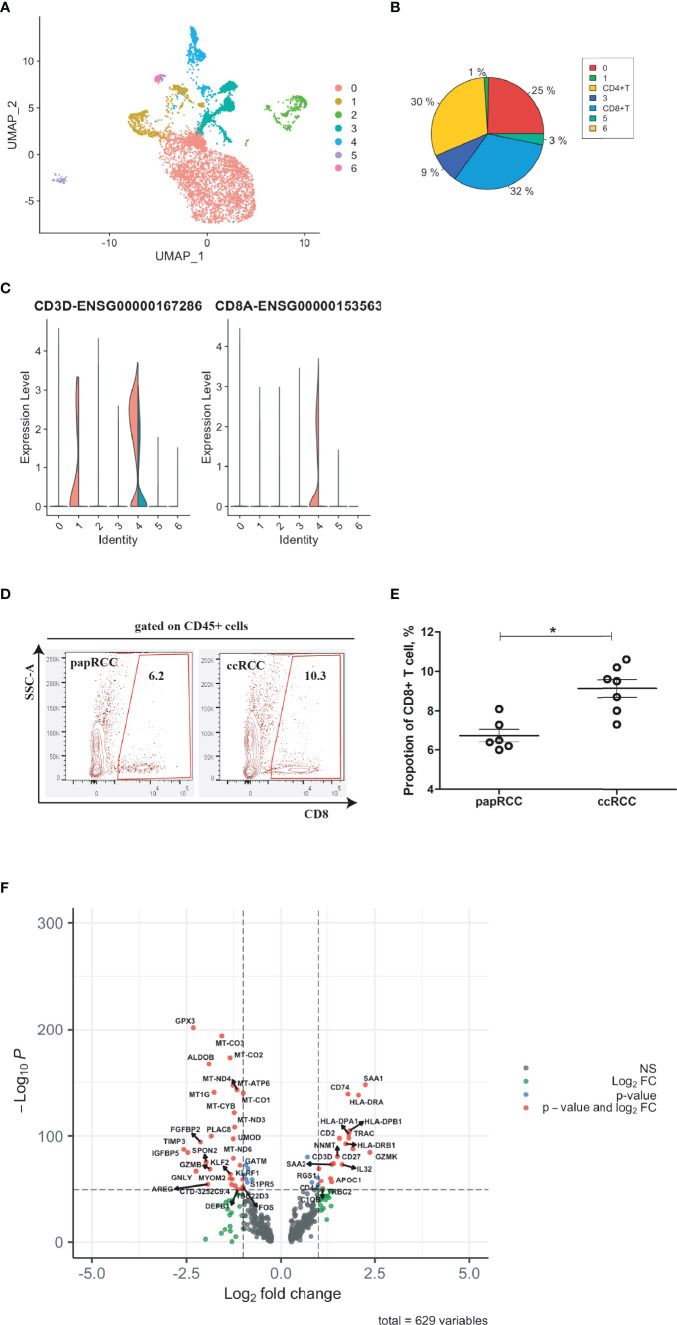
Identification of CD8+ T cell-related genes in clear cell renal cell carcinoma (ccRCC). Single cell RNA (scRNA) data were presented with UMAP clustering using R package Seurat (https://satijalab.org/seurat/v3.1/integration.html). **(A)** UMAP plot showing seven cell clusters in integrated ccRCC and papillary renal cell carcinoma (papRCC) biopsies. **(B)** Pie plot indicating cell proportions in integrated biopsies. **(C)** Combined violin plots showing the comparison of gene expression levels of T cells and CD8+ T cells between ccRCC (red) and papRCC (cyan). **(D)** Flow cytometry analysis of CD8+ T cells in RCC patients. Contour plots showed the frequency of CD8+ T cells in RCC biopsies isolated from patients. **(E)** Dot plot showed statistical analysis of CD8+ T cells in RCC biopsies isolated from patients. Each dot represented one readout. Nonparametric analysis was used. *: p<0.05. **(F)** Volcano plot showing the distribution of 629 differentially expressed genes (DEGs) expressed by CD8+ T cells (ccRCC *vs.* papRCC).

In order to improve prognosis accuracy, CD8+ T cell-related genes were identified by integrating the ccRCC biopsies with the scRNA-seq data of the papRCC group (control) (**Figure 2A**). Seven cell clusters were identified based on a large-scale genetic profile (**Figures 2A, B**). CD8+ T cells (cluster 4) were identified by co-expression of *CD3D* and *CD8A* (**Figure 2C**). From the violin plots, Cluster 1 exhibited significant expression of *CD3D*, which revealed that this could be CD4+ T cells (**Figures 2B, C** and **Supplementary Figure 1**). Furthermore, The isolated CD8+ T cells from scRNA-seq datasets were validated with cancerous biopsies isolated from ccRCC patients and papRCC patients (**Figure 2D**), it seems that the frequency of CD8+ T cells is significantly higher in ccRCC biopsies (**Figure 2E**). For CD8+ T cells, 629 DEGs were identified (**Figure 2F**).

### Construction of a CD8+ T Cell-Related Gene Signature for ccRCC Prognosis

**Figure 3:**
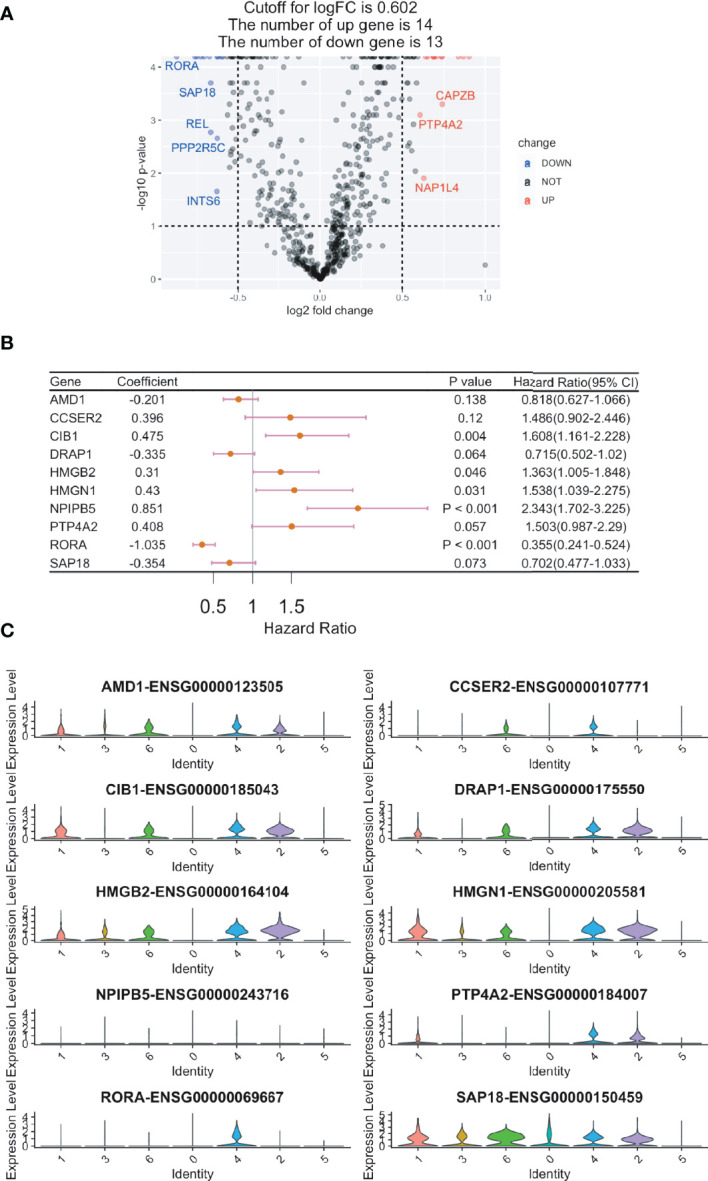
Construction of a CD8+ T cell-related prognostic gene signature for clear cell renal cell carcinoma (ccRCC). Cox regression analysis was used to build a CD8+ T cell-related prognostic gene signature. **(A)** Volcano plot showing the Cox regression analysis results of CD8+ T cell DEGs (ccRCC *vs.* papRCC). DOWN, down-regulated; NOT, not significant; UP, up-regulated. **(B)** Forest plot showing the top ten genes selected by random survival forest algorithm. **(C)** Violin plots showing the expression of the ten genes in different cell types.

Overall, 602 ccRCC cases with bulk RNA sequencing results and clinical features were acquired from the TCGA database. The 629 identified DEGs were subjected to a univariate Cox regression analysis, in which 27 genes were found to be associated with the prognosis of ccRCC (**Figure 3A** and **Supplementary Figure 2**). Using the RSF algorithm, ten genes (i.e., *AMD1, CCSER2, CIB1, DRAP1, HMGB2, HMGN1, NPIPB5, PTP4A2, RORA*, and *SAP18*) were found to be relevant to ccRCC survival rates (**Figure 3B** and **Supplementary Figure 3**). The expression levels of these ten genes are shown in the violin plots (**Figure 3C**).

### Validation of Prognostic Gene Signature for ccRCC

**Figure 4:**
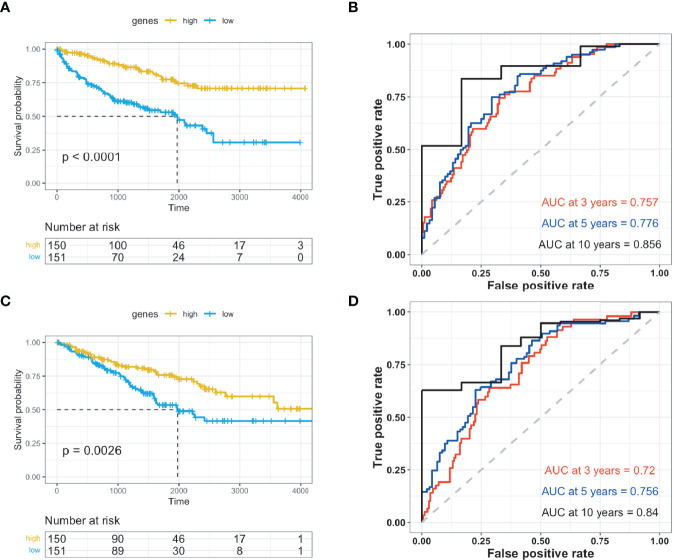
Validation of prognosis genes for ccRCC. The prognostic genes were validated using Kaplan-Meier and receiver operating characteristic studies, and were divided into high- and low-risk groups. **(A)** Kaplan-Meier (KM) investigation of the risk group defined with CD8+ T cell-correlated gene tags in The Cancer Genome Atlas (TCGA) training dataset for ccRCC. **(B)** 3-, 5-, and 10-year receiver operating characteristic (ROC) curves for TCGA training dataset of ccRCC. **(C)** Kaplan-Meier (KM) investigation of the risk group defined with CD8+ T cell-correlated gene tags in the TCGA testing dataset for ccRCC. **(D)** 3-, 5-, and 10-year ROC curves for TCGA testing dataset of ccRCC.

The ten identified genes were used to build a risk scoring system by employing multivariate Cox analysis to the 602 ccRCC cases obtained from the TCGA database. The risk score for each case was calculated accordingly. Patients with ccRCC were classified into either high- or low-risk groups based on optimized cut-off values. In the high-risk group, patients exhibited higher overall survival rates in both the training and testing groups (**Figures 4A, C** and **Supplementary Figure 4**). The predictive power of the genes was assessed, and ROC curves calculated from the ccRCC cases were plotted for both the training and testing groups (**Figures 4B–D** and **Supplementary Figure 5**–**7**).

### Relationship Between Risk Scores and Clinical Features in ccRCC

**Figure 5:**
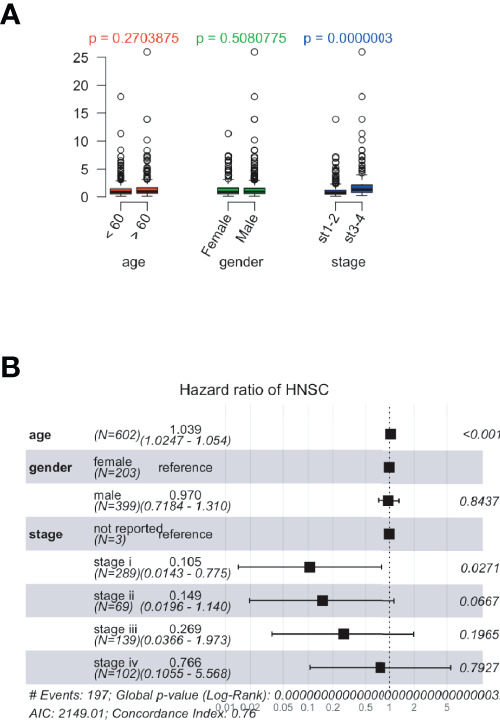
The relationship between risk scores and clinical features in The Cancer Genome Atlas (TCGA) database. The risk scores were calculated based on the correlation between identified prognostic genes and patient’s clinical data. **(A)** Dot plots presenting the risk score distribution of age, sex, and clinical stages in the TCGA database. **(B)** Forest plots showing risk scores and clinical features in TCGA dataset.

Using data from the TCGA database, patients with ccRCC were divided into high- or low-risk groups with optimized cut-off values. Box plots showed that age and sex were irrelevant to prognosis, and clinical stages of ccRCC correlated with prognosis (**Figure 5A**). Moreover, these factors (e.g., age, sex, clinical stage, and pathological features) were assessed for their contribution towards the prognosis. Forest plots showed that these factors correlated with the risk group results (**Figure 5B**). Additionally, genetic features showed a notable significance compared to the other factors.

### SAP18 Expressing CD8+ T Cells Colocalize With Neutrophils in ccRCC Biopsies

**Figure 6:**
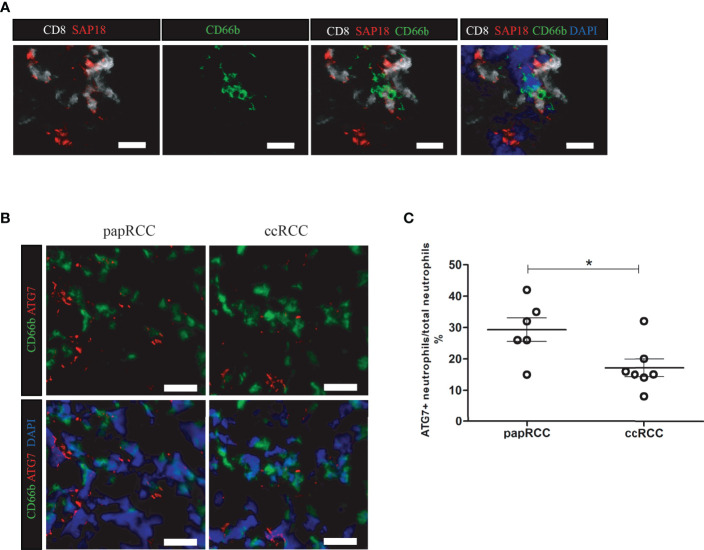
SAP18-expressing CD8+ T cells co-localize with neutrophil in ccRCC biopsies. **(A)** Immunofluorescent imaging showing CD8+ T cells in ccRCC biopsies expressing SAP18, and co-localize with neutrophils. CD8+ T cells showing in white, SAP showing in red, neutrophils were labeled with CD66b (green), cell nucleus were counterstained with DAPI. White scale bar indicates 25 μm. **(B)** Immunofluorescent imaging Neutrophils (CD66b+ cell, green) in ccRCC expressing less ATG7 (red). cell nucleus were counterstained with DAPI. White scale bar indicates 25 μm. **(C)** Statistical analysis of the frequency of ATG7+ expressing neutrophils against total neutrophils in RCC biopsies. Each dot represented one readout. *: p<0.05. Nonparametric analysis was used.

It has been reported that SAP18 among those four potential prognostic biomarkers expressed by CD8+ T cells, SAP18 has been reported to be critical for autophagy (13, 14), a manner for immune system cleaning out aged cells or injured cells. In tumor microenvironment, neutrophil metabolic function in autophagy and free fatty acid is considered to be important for malignancy (15, 16). In ccRCC biopsies, SAP18-expressing CD8+ T cells were observed frequently to co-localize with neutrophils (**Figure 6A**). Furthermore, its’s reported that ATG7 is essential for neutrophil metabolic function, especially in autophagy for providing free fatty acid (17). Comparing with papRCC, neutrophils in ccRCC biopsies exhibited obviously decreased expression of ATG7 (**Figures 6B, C**), indicating neutrophil metabolic function in ccRCC were inhibited.

## Discussion

Previous studies have reported the presence of infiltrated CD8+ T cells in ccRCC biopsies, which could help determine the subsets of ccRCC (18, 19). It has been shown that the frequency of a specific subpopulation of CD8+ T cells predicts the survival of patients with ccRCC (20), however, few studies have identified the related genes/proteins. With the advent of scRNA-seq and bulk-seq data (21) in combination with clinical features, it was possible for us to explore genetic profiles in a large-scale manner, and identify potential genes that could be used as prognostic biomarkers.

Among the infiltrated CD8+ T cells, ten genes were identified to be related to ccRCC prognosis and six were reported to be valuable in predicting cancer prognosis; including *AMD1* for assessing patients with gastric cancer (22), *CIB1, PTP4A2*, and *HMGB2* for breast cancer (23–26), *HMGN1* for leiomyosarcoma (27), and *RORA* for glioma (28). It has been reported that increased expression level of *AMD1* in gastric cancer indicates up-regulated enzymatic function in synthesis of spermidine or spermine (22), which could possibly promote the development of cancer; it has been frequently observed that HMGB2 is expressed in malignant gastric cancer (29) and in breast cancer (25), playing a role in modulation *via* targeting LDHB and FBP1 (25).

Another merit of the present study is that it emphasized the importance of these six genes in the prediction of ccRCC patient survival by analyzing the large-scale genetic profile from isolated, infiltrated CD8+ T cells, together with clinical features from long-term cohort studies. Moreover, we identified four genes (*CCSER2, DRAP1, NPIPB5*, and *SAP18*) related to ccRCC patient survival.

Undoubtedly, CD8+ T cells and neutrophils exert intermodulation phenomena. It has been reported that in the tumor microenvironment, low-density neutrophils could suppress CD8+ T cell proliferation in peripheral blood (30), and could promote the apoptosis of non-activated CD8+ T cells. On the other hand, CD8+ T cell could also modulate neutrophil-mediated immunosuppression *via* RANK signals (31). It has been reported that neutrophils could form extracellular traps, with which drive CD8+ T cell exhibited a metabolic exhausted phenotype (32). In this study, however, we showed that CD8+ T cell could potentially influence neutrophil’s metabolism in ccRCC.

The potential protective role of CD8+ T cells in ccRCC could be induced by altered expression of SAP18, compared with papRCC. SAP18 has been shown to modulate neutrophil migration and autophagy in tumor microenvironment (33); meanwhile neutrophil autophagy is highly related to the development of tumor (16). Both neutrophil migration and autophagy requires the metabolism of free fatty acid, in which ATG7 plays a critical role (17). The observation of decreased expression of ATG7 in neutrophils in ccRCC biopsies suggested altered neutrophil metabolism, and provides an explanation that CD8+ T cells could potentially modulate neutrophil metabolism of free fatty acid by providing SAP18.

The results obtained from this study should be considered in light of certain limitations. For example, the data and experiments used in this study were obtained from cross-sectional studies, the conclusion draw from such studies should be validated with prospective studies, or with studies based on genetic modified animals. The RCC patients who provided biopsies were volunteered from a single site, thus the results could be less applicable to different populations.

## Conclusions

Overall, this study identified ten potential genes that could serve as prognostic biomarkers for patients with ccRCC and connect the bridge between the role of the neutrophil metabolism and the behavior of renal cell carcinoma.

## Data Availability Statement

The datasets presented in this study can be found in online repositories. The names of the repository/repositories and accession number(s) can be found in the article/**Supplementary Material**.

## Ethics Statement

The studies involving human participants were reviewed and approved by Youjiang Medical University for Nationalities. The patients/participants provided their written informed consent to participate in this study.

## Author Contributions

LM and HL designed this study. JW, FH, JZ, and PH performed analysis of the scRNA-seq data. JT, MH, RM, and YX performed analysis of the bulk sequencing data. SH, ZW, and JS performed Immunofluorescent staining. All authors contributed to the article and approved the submitted version.

## Funding

This research was funded by grants from Guangxi Natural Science Foundation (#2020GXNSFAA259050 and #2020GXNSFAA259081), from Youjiang Medical University for Nationalities (#yy2019bsky001), from the Research and Development of Appropriate Medical Technology in Guangxi (S2017082), Guangxi Key Research and Development Plan (AB17195005) from High-Level Talent Research Projects of the Affiliated Hospital of Youjiang Medical University for Nationalities (#R20196341), and from Self-Raised Project of Baise (#20171111).

## Conflict of Interest

The authors declare that the research was conducted in the absence of any commercial or financial relationships that could be construed as a potential conflict of interest.

## Publisher’s Note

All claims expressed in this article are solely those of the authors and do not necessarily represent those of their affiliated organizations, or those of the publisher, the editors and the reviewers. Any product that may be evaluated in this article, or claim that may be made by its manufacturer, is not guaranteed or endorsed by the publisher.
